# Derepressing *muscleblind* expression by miRNA sponges ameliorates myotonic dystrophy-like phenotypes in *Drosophila*

**DOI:** 10.1038/srep36230

**Published:** 2016-11-02

**Authors:** Estefania Cerro-Herreros, Juan M. Fernandez-Costa, María Sabater-Arcis, Beatriz Llamusi, Ruben Artero

**Affiliations:** 1 Translational Genomics Group, Incliva Health Research Institute, Valencia, Spain; 2 Department of Genetics and Interdisciplinary Research Structure for Biotechnology and Biomedicine (ERI BIOTECMED), Universitat de València, Valencia, Spain

## Abstract

Myotonic Dystrophy type 1 (DM1) originates from alleles of the *DMPK* gene with hundreds of extra CTG repeats in the 3′ untranslated region (3′ UTR). CUG repeat RNAs accumulate in foci that sequester Muscleblind-like (MBNL) proteins away from their functional target transcripts. Endogenous upregulation of MBNL proteins is, thus, a potential therapeutic approach to DM1. Here we identify two miRNAs, *dme-miR-277* and *dme-miR-304*, that differentially regulate *muscleblind* RNA isoforms in miRNA sensor constructs. We also show that their sequestration by sponge constructs derepresses endogenous *muscleblind* not only in a wild type background but also in a DM1 *Drosophila* model expressing non-coding CUG trinucleotide repeats throughout the musculature. Enhanced *muscleblind* expression resulted in significant rescue of pathological phenotypes, including reversal of several mis-splicing events and reduced muscle atrophy in DM1 adult flies. Rescued flies had improved muscle function in climbing and flight assays, and had longer lifespan compared to disease controls. These studies provide proof of concept for a similar potentially therapeutic approach to DM1 in humans.

Myotonic dystrophy type 1 (DM1) is an incurable neuromuscular disorder that is caused by an expanded CTG*CAG repeat in the 3′-untranslated region (3′ UTR) of the *dystrophia myotonica-protein kinase* (*DMPK*) gene (for a recent review, see ref. 1). The normal human *DMPK* gene harbors 5–37 copies of the trinucleotide motif, but a dynamic mutation may increase this number to over 5000 repeat copies. Clinically, DM1 is a multisystemic disorder, which mainly affects skeletal muscle, the heart and the nervous system. Severity of disease correlates with the expansion size and typical disease features are myotonia, muscle weakness and atrophy, smooth and cardiac muscle involvement, CNS dysfunction, somnolence, endocrine disorders and reduced life span^2^,^3^.

Expression of expanded alleles in DM1 results in the nuclear retention of mutant *DMPK* mRNA and reduced DMPK protein levels^4^. Mutant transcripts sequester Muscleblind-like (MBNL) splicing factors, leading to the abnormal alternative splicing of a multitude of other transcripts and the expression of fetal forms of their protein products in DM1 adults^5^,^6^,^7^. Spliceopathy is therefore thought to be the major factor underlying the pathogenesis of DM1. However, alternative mechanisms such as additional changes in gene expression, antisense transcripts, translation efficiency, misregulated alternative polyadenylation and miRNA deregulation may also contribute to the pathogenesis of DM1^8^,^9^,^10^,^11^,^12^,^13^,^14^,^15^.

Several therapeutic approaches have been tested in DM1 animal models. Among them, the most exciting results derived from blocking the interaction between MBNL and toxic RNA using small molecules, peptides, morpholinos or antisense oligonucleotides, and gapmers to degrade the mutant transcripts^16^,^17^,^18^,^19^,^20^,^21^. A less explored alternative in DM1 is the therapeutic modulation of MBNL gene expression. Although the expression of CUG expansions triggers different molecular alterations, current evidence points to MBNL depletion as the main cause of disease symptoms. A *Mbnl1* knock-out (KO) mouse model displays myotonia, missplicing of muscular transcripts and cataracts, which are all characteristic symptoms of DM1 disease^22^. More recently, relevant cardiac dysfunction features have been described in 2 month-old *Mbnl1* mutant mice (hypertrophy, interstitial fibrosis and splicing alterations), which suggests a role for Mbnl1 reduction in the cardiac problems in DM1^23^. However, *Mbnl1* KO mice do not display the whole set of symptoms of DM1. Therefore, it has been hypothesized that *Mbnl2* could compensate for *Mbnl1* loss of function in these mice. In fact, *Mbnl1* KO mice with reduced expression of *Mbnl2* (*Mbnl1*^−/−^; *Mbnl2*^+/−^), are viable but develop most of the cardinal defects of the disease, including reduced lifespan, cardiac blockage, severe myotonia, atrophic fibers and progressive weakness of skeletal muscles. In support of the compensation hypothesis, levels of *Mbnl2* are increased in *Mbnl1*^−/−^ KO mice and Mbnl2 can regulate the splicing of exons which are normally regulated by Mbnl1^24^. Furthermore, genetic polymorphisms in the human MBNL1 gene promoter have been associated with the severity of the disease^25^.

Several observations suggest that MBNL1 overexpression has potential for treating DM1 pathology. Firstly, administration of recombinant Mbnl1 protein to a *HSA*^*LR*^ mouse model of DM1, rescues myotonia and the splicing alterations characteristic of DM1^26^. Secondly, we showed that the overexpression of a *muscleblind* isoform partially rescues muscle atrophy in a *Drosophila* DM1 model^27^. Finally, MBNL1 overexpression is well tolerated in skeletal muscle in transgenic mice where it causes only relatively minor splicing changes but no effect on longevity^28^.

In this proof of concept study, we use the *Drosophila* DM1 model to explore the therapeutic potential of silencing specific microRNAs (miRNAs) and thus boost *muscleblind* (*mbl*) expression. The fundamental roles of miRNAs in the regulation of gene expression have been well-established. These endogenous ∼22 nucleotide-long non-coding RNAs act post-transcriptionally and exert their regulatory effects mainly by binding to the 3′ UTR of target mRNAs, which results in mRNA deadenylation and decay, translational suppression or, rarely, mRNA cleavage^29^,^30^,^31^,^32^,^33^. Starting from a set of miRNAs predicted as *muscleblind* regulators, we confirmed that specific silencing of two of them, using sponge constructs, which sequester the miRNAs, upregulated *muscleblind* mRNA and protein. Similar effects were observed in flies co-expressing 480 CTG interrupted repeats (*i(CTG)480*) and either of the two sponge constructs in muscle. Muscleblind upregulation was sufficient to rescue characteristic DM1 model phenotypes such as missplicing events, reduced lifespan, and muscle atrophy. Importantly, the rescue of muscle atrophy resulted in improved climbing and flight ability in DM1 model flies. These data provide proof-of-principle for the therapeutic potential of Muscleblind upregulation by specific miRNA inhibitors in DM1 patients.

## Results

### Silencing of *dme-miR-277* or *dme-miR-304* derepresses *muscleblind* in *Drosophila* muscle

Muscleblind sequestration in RNA foci and subsequent loss of function of the protein is a main triggering factor in DM1 molecular pathology. In order to identify miRNAs that repress *muscleblind* we selected candidate miRNAs and blocked their activity using specific miRNA sponges. We selected *dme-miR-92a*, *dme-miR-100* and *dme-miR-124* based on data generated in our laboratory and their orthology with human miRNAs. To widen the search of miRNA set of candidates, we used TargetScan^34^ to analyze miRNA recognition sites in the *muscleblind* 3′UTR and identified sites for two additional miRNAs: *dme-miR-277* and *dme-miR-304* (Table 1). Importantly, profiling of *Drosophila* microRNA expression in dissected thoracic muscles, had previously demonstrated *miR-124, miR-100, miR-277* and *miR-304* expression in these muscles^35^.

**Figure 1:**
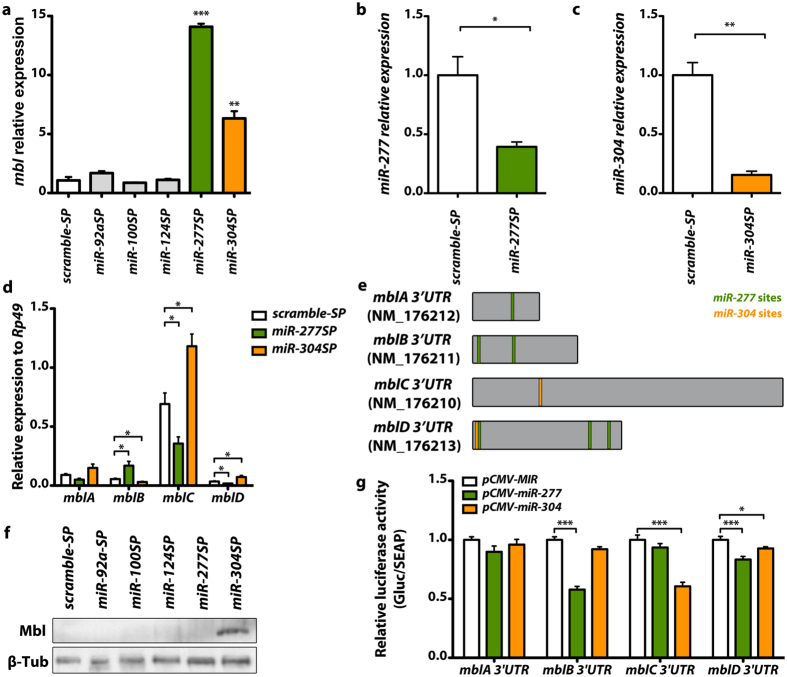
Tissue-specific silencing of *dme-miR-277* and *dme-miR-304* upregulates *muscleblind* mRNA and protein in *Drosophila* muscle. (**a**) qRT-PCR amplification of *muscleblind* from flies expressing miRNA sponge constructs for *dme-miR-92a*, *dme-miR-100*, *dme-miR-124*, *dme-miR-277* and *dme-miR-304* in muscle. *muscleblind* expression levels were strongly upregulated in *miR-277SP* and *miR-304SP* flies. (**b**) Analysis of the levels of *muscleblind* isoforms by qRT-PCR. *dme-miR-277* silencing in muscle caused an upregulation of the *mblB* isoform whilst expression levels of the *mblC* and *mblD* isoforms were reduced in *miR-277SP* flies. Conversely, *mblC* and *mblD* levels were increased and the *mblB* isoform was reduced in *miR-304SP* flies. (**c**) Detection of Muscleblind protein by Western blot. An increase of Muscleblind protein was only detected in *miR-304SP* flies. All the indicated transgenes were driven in muscle using *Mhc-Gal4.* Histogram showing *dme-miR-277* (**d**) and *dme-miR-304* (**e**) relative expression levels according to qRT-PCR data. Both miRNAs were significantly silenced in flies expressing the corresponding sponge constructs under the control of *Mhc-Gal4* compared to flies that expressed *scramble-SP* (control). (**f**) Scheme of the predicted binding sites for *dme-miR-277* and *dme-miR-304* in *muscleblind* 3′ UTRs (*mblA* to *mblD*). Reference sequence accessions and size (in nt) are also included. Representation is to scale. (**g**) Quantification of Gaussian luciferase activity relative to alkaline phosphatase (Gluc/SEAP) of HeLa cells cotransfected with the indicated *mbl* 3′ UTR sensor constructs and plasmids expressing *dme-miR-277* or *dme-miR-304*. Significantly reduced relative luminescence compared to empty vector (pCMV-MIR, control) reveals direct binding of *dme-miR-277* to m*blB* and m*blD* 3′UTRs and of *dme-miR-304* to m*blC* and m*blD* 3′ UTRs. The graphs show means±s.e.m. *p < 0.05, **p < 0.01, ***p < 0.001 (Student’s t-test).

To validate that these miRNAs regulate Muscleblind, we targeted the expression of miRNA sponge constructs^35^, *UAS-miR-XSP*, to the *Drosophila* muscles using the *Myosin heavy chain* (*Mhc*)*-Gal4* driver line and analyzed *muscleblind* transcript levels by qRT-PCR. We used specific primers to amplify a region in *muscleblind* exon 2, which is shared by all known transcript isoforms^36^,^37^. As a control, we used a scramble miRNA sponge line (*UAS-scramble-SP*). No significant increase in *muscleblind* expression level was detected in flies expressing *miR-92aSP*, *miR-100SP* or *miR-124SP* under the control of *Mhc-Gal4*. In contrast, *muscleblind* transcripts were significantly increased in flies that expressed *miR-277SP* or *miR-304SP* in muscle compared with *scramble-SP* controls (Fig. 1a). *Muscleblind* levels were 14-fold higher when *dme-miR-277* was inhibited while *dme-miR-304* silencing resulted into a 6-fold increase. Consistently, the quantification of the expression of the mCherry reporter contained in the SP constructs showed that *miR-277*SP and *miR-304SP* were the two SPs with the highest expression in the flies (Fig. S1). Thus, we cannot disprove that *miR-92aSP*, *miR-100SP* or *miR-124SP* regulate *muscleblind* since their SP constructs had comparatively lower expression levels. As we were not interested in a complete description of *muscleblind* regulation by microRNAs but in providing proof of concept of their usefulness as therapeutic targets in DM1, we continued our studies with the two confirmed *muscleblind* regulators; *miR-277* and *miR-304.* To assess the efficiency of miRNA downregulation by driving sponge constructs with the *Mhc*-Gal4 driver, we performed qRT-PCRs to detect the levels of the corresponding RNAs and confirmed that flies expressing *miR-277SP* or *miR-304SP* had reduced levels of the corresponding microRNA (Fig. 1b,c). *dme-miR-277* was silenced ~60% while a robust reduction of ~80% was detected for *dme-miR-304.* Therefore, these results demonstrate that silencing of *dme-miR-277* or *dme-miR-304* derepresses *muscleblind*.

### dme-miR-277 and dme-miR-304 regulate different Muscleblind isoforms

*Drosophila muscleblind* is a large gene, spanning more than 110 kb, which gives rise to different 3′ UTRs through the use of alternative 3′ exons^36^,^37^. Experimental evidence suggests that *muscleblind* isoforms are not functionally redundant^38^. To determine which *muscleblind* isoforms are regulated by *dme-miR-277* or *dme-miR-304*, we used the Miranda algorithm^39^ to identify *dme-miR-277* and *dme-miR-304* recognition sites in Muscleblind isoform 3′ UTRs (Table 1, Fig. 1e). Importantly, note that Miranda database allowed the search in *mblA*, *mblB*, *mblC* and *mblD* transcripts named according to^36^ but did not include the recently identified isoforms *mblH*, *mblH′*, *mblJ* and *mblK*^37^. We found one potential recognition site for *dme-miR-277* in the *mblA* isoform and two in *mblB* and *mblD*. qRT-PCR analyses revealed that the level of *mblB* significantly increased when *dme-miR-277* was inhibited. *mblD* expression levels were reduced in *Mhc-Gal4 miR-277SP* flies and no significant differences were detected for *mblA* compared with *scramble-SP* control flies (Fig. 1d). Intriguingly, expression level of *mblC*, an isoform with no predicted recognition sites for *dme-miR-277,* were significantly reduced in *Mhc-Gal4 miR-277SP* flies. For *dme-miR-304*, we found one recognition site in the *mblC* and *mblD* 3′ UTR and observed a significant upregulation of both isoforms in *Mhc-Gal4 miR-304SP* flies (Fig. 1d). Notably, silencing of *dme-miR-304* in muscle triggered a strong increase in the level of *mblC*, which is the most expressed isoform in adult flies^38^. The fact that *dme-miR-277* and *dme-miR-304* silencing cause isoform-specific changes in *muscleblind* expression levels suggest that these miRNAs directly regulate the *mbl* transcripts. To confirm direct binding of these miRNAs to the corresponding 3′ UTRs of *mbl* transcripts, we performed luciferase reporter gene assays in HeLa cells. In these studies, the 3′ UTR of the different *mbl* transcripts were cloned downstream of Gaussia luciferase and the interaction of the microRNAs to their targets in these regions, was detected as a decrease in the luminescence measurements. These experiments confirmed direct binding of *dme-miR-277* to the 3′ UTR of the *mblB* and *D* isoforms and direct binding of *dme-miR-304* to *mbl* isoforms C and D (Fig. 1e,g).

**Figure 2:**
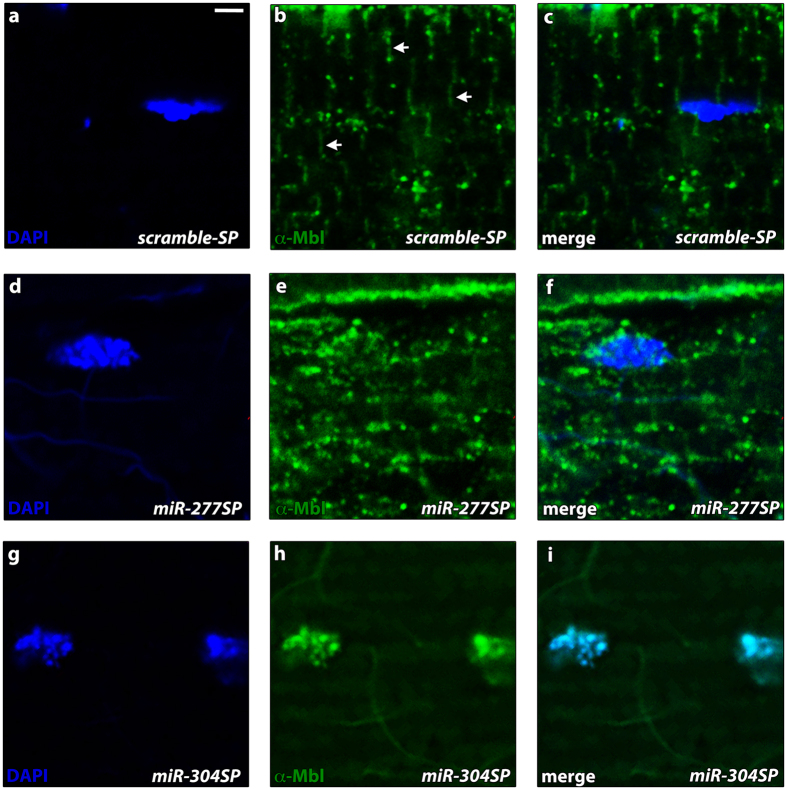
*dme-miR-277* and *dme-miR-304* silencing upregulates Muscleblind proteins with different subcellular localization. Representative confocal images of longitudinal sections of IFMs showing anti-Mbl staining (green). Nuclei were counterstained with DAPI (blue) (**a**–**c**) Endogenous Muscleblind expression was preferentially detected in sarcomeric bands while a low signal was detected in some cell nuclei. Arrow-heads in (**b**) point to the sarcomeric bands. (**d–f**) An increase in the Muscleblind cytoplasmic signal was detected in *miR-277SP* flies. In contrast, silencing of *dme-miR-304* in IFMs boosted the Muscleblind nuclear signal (**g**–**i**). Scale bar = 2 μm.

Given that miRNAs can act either by reducing target transcript levels or blocking their translation, we decided to analyze Muscleblind protein levels to validate the regulatory miRNA candidates. With this aim, we used an anti-Mbl antibody that has previously been optimized to detect overexpression of MblA, MblB and MblC protein, but not their endogenous expression^40^,^41^. Western blotting analyses revealed an increase in Muscleblind protein levels only in *Mhc-Gal4 miR-304SP* flies (Fig. 1f). Consistently with the qRT-PCR determinations, we only detected one band in the Western blot corresponding to MblC protein. To further analyze the effect of *dme-miR-277* or *dme-miR-304* silencing we stained Muscleblind distribution in longitudinal sections of indirect flight muscles (IFMs). We had previously shown that endogenous Muscleblind protein is localized mainly in sarcomeric Z and H bands of muscle^42^. Consistently, we detected Muscleblind proteins in the bands of muscle sarcomeres in control flies that express the *scramble-SP* construct (Fig. 2a–c). Interestingly, *dme-miR-277* and *dme-miR-304* exhaustion, had different effects on Mbl protein distribution. *dme-miR-277* silencing increased cytoplasmic Mbl, (Fig. 2d–f), while a strong nuclear localization was detected in *Mhc-Gal4 miR-304SP* flies (Fig. 2g–i). Taken together, these results demonstrate that endogenous Muscleblind isoforms can be upregulated by blocking *dme-miR-277* and *dme-miR-304* inhibitory activity.

### dme-miR-277 or dme-miR-304 silencing upregulates Muscleblind expression in a *Drosophila* DM1 model

**Figure 3:**
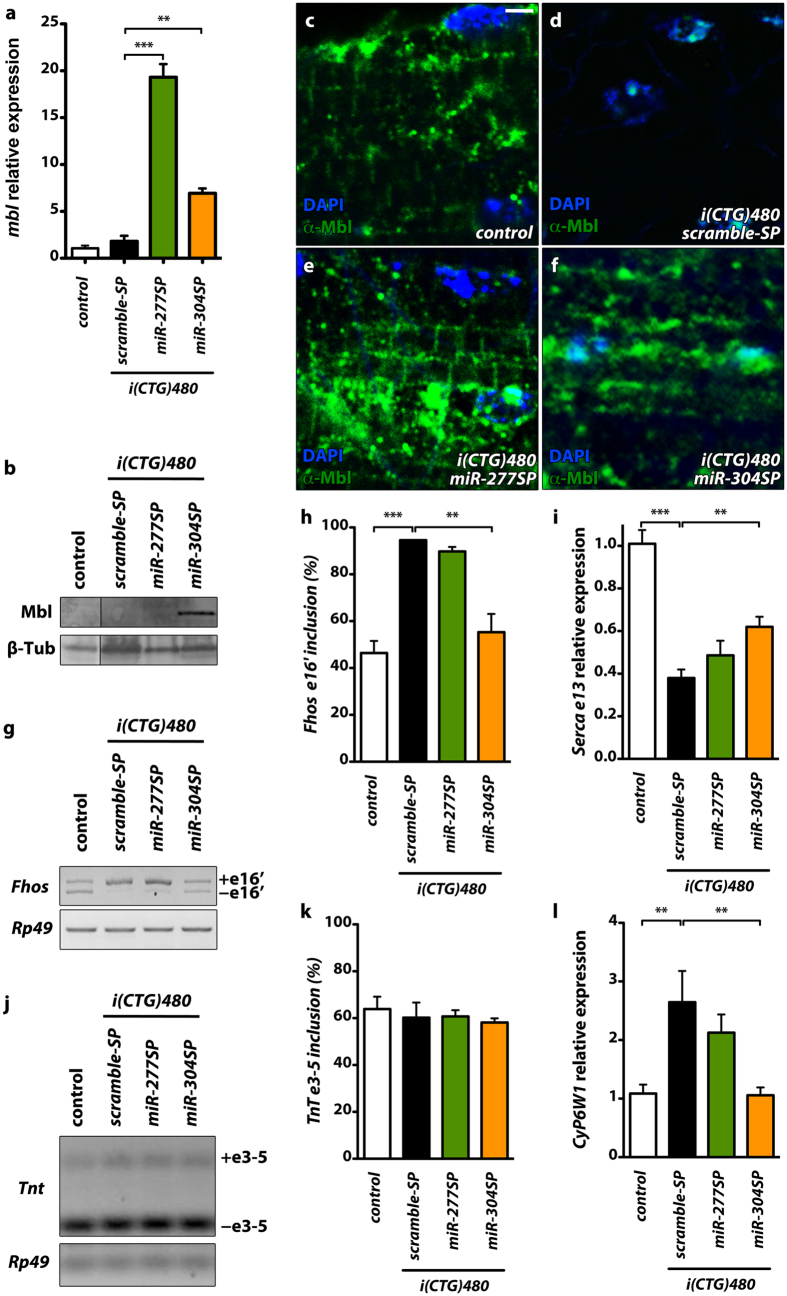
*dme-miR-277* or *dme-miR-304* silencing enhance *muscleblind* expression and rescues missplicing events in a DM1 background. (**a**) Bar graph showing *muscleblind* expression levels qRT-PCR data. *muscleblind* mRNA was significantly upregulated in model flies expressing *miR-277SP* and *miR-304SP* in comparison to flies that did not express the expansions (control, *Mhc-Gal4*/*+*) or model flies expressing *scramble-SP*. (**b**) Western blot analysis showed additional Muscleblind C only in model flies expressing *miR-304SP*. (**c–f**) Confocal images of longitudinal sections of IFMs reveal Muscleblind (green) distribution to sarcomeric bands in control flies (**c**). In contrast, Muscleblind was found in nuclear aggregates in IFMs expressing CTG expansions (**d**). Expression of *miR-277SP* in model flies released Muscleblind from aggregates and restored its distribution to sarcomeric bands (**e**). *miR-304SP* expression achieved a dispersed overexpression of Muscleblind in both nuclei and cytoplasm (**f**). Nuclei were counterstained with DAPI (blue). (**g**) RT-PCR to assess inclusion of *Fhos* exon 16′ in flies with different genotypes. *Rp49* transcripts were detected as endogenous control. (**h**) Quantification of percentage of exon inclusion (according to g) confirmed an improvement of *Fhos* missplicing in model flies expressing *miR-304SP*. (**i**,**l**) qRT-PCR results of *Serca* exon 13 and *CyP6W1* expression relative to *Rp49*, confirmed a significant rescue of both events in model flies expressing *miR-304SP*. (**j**) RT-PCR showing inclusion of *TnT* exon 3–5, which did not differ in the studied genotypes. (**k**) Quantification of exon percentage inclusion according to (**j**). All the indicated genotypes were driven to muscle using *Mhc-Gal4*. Scale bar = 2 μm. *p < 0.05, **p < 0.01, ***p < 0.001 (Student’s t-test).

Previous *Drosophila* models of DM1 displayed ribonuclear foci in muscle cells containing Muscleblind proteins^43^,^44^,^45^. To test the effect of specific silencing of the miRNA repressors of *muscleblind* in a *Drosophila* DM1 model, we studied Muscleblind expression in flies expressing 480 interrupted CTG repeats under the control of the muscle-specific driver Myosin heavy chain with simultaneous expression of sponge constructs (*Mhc-Gal4 UAS-i(CTG)480 UAS*-*miR-XSP*). Analyses of *muscleblind* transcript levels by qRT-PCR showed that silencing of *dme-miR-277* or *dme-miR-304* upregulated *muscleblind* in DM1 flies (Fig. 3a). *muscleblind* transcript levels were 19-fold higher in flies expressing both *i(CTG)480* and *miR-277SP* and 7-fold higher in those expressing *i(CTG)480* and *miR-304SP* compared to controls. Moreover, in agreement with protein analyses in *miR-SP* (Fig. 1c), silencing of *dme-miR-304* triggered an increase of MblC protein levels in DM1 flies (Fig. 3b).

To study the effect of *dme-miR-277* or *dme-miR-304* silencing on Muscleblind subcellular localization in DM1 flies, we examined Muscleblind protein distribution by inmunodetection in IFMs. Expression of either *miR-277SP* or *miR-304SP* in DM1 model flies released Muscleblind from ribonuclear foci and increased the level of protein, both in nuclei and in cytoplasm (Fig. 3c–f). In the case of model flies expressing *miR-277SP*, Muscleblind distribution in sarcomeric bands of muscle, which is characteristic of control flies not expressing the repeats, was significantly rescued. Similarly, expression of *miR-304SP* led to a detectable increase of Muscleblind dispersed in nuclei and cytoplasm. Of note, in flies that did not express the CTG repeats, *dme-miR-304* silencing results in increase of Muscleblind only in nuclei (compare Figs 2i and 3f). The overall greater derepression of *mbl* in a CTG background, compared to wild type, may stem from the higher transcription or stability of *muscleblind* transcripts, perhaps as a compensatory mechanism, in DM1 flies (1.8 fold; Fig. 3a). Hence, when exposed to the same levels of *miR-304SP* sponge, more muscleblind transcripts may be available to translation in a DM1 background. Therefore, silencing of *dme-miR-277* or *dme-miR-304* upregulates Muscleblind levels and rescues its subcellular distribution in DM1 fly muscles.

### *dme-miR-304* silencing rescues molecular defects in a *Drosophila* DM1 model

RNA metabolism alterations are the major biochemical hallmark in DM1. Specifically, spliceopathy is the only molecular alteration that has been directly linked with DM1 symptoms. To test whether Muscleblind increase, triggered by *dme-miR-277* or *dme-miR-304* silencing, was enough to rescue missplicing in the DM1 model flies we studied two altered splicing events (Fig. S2g) and the alteration in the expression level of a specific transcript. First, we identified *Fhos* exon 16′ missplicing in DM1 flies and demonstrated that this splicing event and Serca exon 13 inclusion, previously identified as altered in the *Drosophila* DM1 model, both are regulated by Muscleblind C (Fig. S2a,b)^45^. Second, we confirmed that the expression level of the *CyP6W1* gene was also dependent on *mblC* expression (Fig. S2c,f). In DM1 model flies, we confirmed a 2-fold increase of *Fhos* exon 16′ inclusion, a 2.4-fold reduction of *Serca* transcripts with exon 13 and a 3-fold increase of *CyP6W1* expression in comparison to control flies not expressing the repeats. Expression of *miR-304SP* in these flies achieved a complete rescue of *Fhos* splicing and *CyP6W1* expression and a significant 20% increase of *Serca* transcripts including exon 13 (Fig. 3g–i,l). Notably, *dme-miR-304* silencing in muscle caused a strong increase in the level of *mblC* (Fig. 3b), which is an isoform previously shown to act as splicing regulator^38^. Conversely, expression of *miR-277SP*, which rescued Muscleblind expression in cytoplasm, and reduced *mblC* expression levels, did not modify these splice events. As a control, we confirmed that the splicing pattern of *Tnt* exons 3–5, which is not altered in DM1 adult flies^44^, was neither modified by the expression of the sponge constructs nor by *mbl* expression alterations (Fig. 3j,k; Fig. S2d,e). These results show that the level of *muscleblind* derepression achieved with miRNA sponges is enough to trigger significant molecular rescues.

### *dme-miR-277* or *dme-miR-*304 silencing rescues muscle atrophy and motor function in a *Drosophila* model of DM1

**Figure 4:**
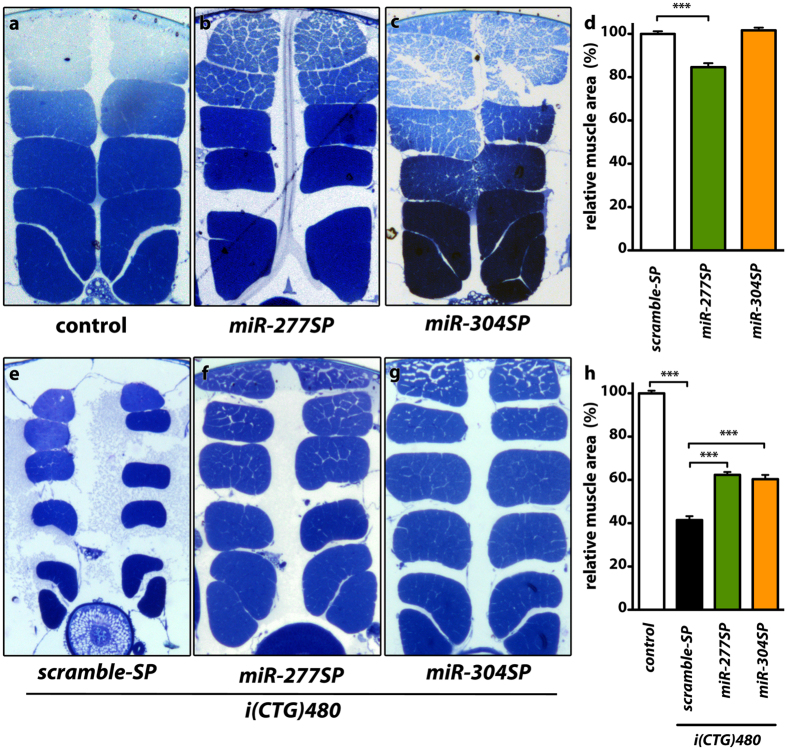
*dme-miR-277* or *dme-miR-304* silencing rescue muscle atrophy in model flies. (**a**–**c**,**e–g**) Representative dorsoventral sections of resin-embedded thoraces of flies with the indicated relevant genotypes. Compared to control flies (**a**) muscle-specific expression of *miR-277SP* resulted into a significant reduction of indirect flight muscle (IFM) area (**b**) whereas *miR-304SP* expression had no effect on this phenotype (**c**) In DM1 model flies the IFM muscle area was reduced to 40% of normal (**e**) In model flies co-expressing either *miR-277SP* or *miR-304SP* and *i(CTG)480* the muscle area increased to 60% of normal (**f**,**g**). (**d**,**h**) Quantification of the mean percentage of muscle area per genotype. The graphs show means ± s.e.m. All the indicated genotypes were driven to muscle using *Mhc-Gal4*. *p < 0.05, **p < 0.01, ***p < 0.001 (Student’s t-test). In all images the dorsal side is on top.

To assess the functional relevance of Muscleblind increase achieved by the expression of specific sponge constructs, we studied the effect of *dme-miR-277* or *dme-miR-304* silencing on muscle atrophy, which is a characteristic alteration in DM1 individuals. To study muscle atrophy we first measured muscle area in dorsoventral sections of IFMs in control flies expressing either *miR-277SP* or *miR-304SP* in muscle (Fig. 4a–d). *dme-miR-277* inhibition induced a reduction of 15% in IFM area, in comparison to flies expressing *scramble-SP* as control. Importantly, *miR-304SP* expression had no effect on this parameter. We have previously reported muscle atrophy in *i(CTG)480* flies^27^,^44^. In these DM1 model flies, we found that tissue-specific silencing of *dme-miR-277* or *dme-miR-304* was enough to rescue muscle area percentage significantly (Fig. 4e–h). In comparison to control flies that did not express the CUG repeats, the mean area of IFMs in model flies expressing the *scramble-SP* was significantly reduced to 40%. Concomitant expression of CUG repeats and either *miR-277SP* or *miR-304SP* resulted in a 20% increase of muscle area in these flies. These data confirm that derepression of *muscleblind* by miRNA silencing was sufficient to rescue muscle atrophy in *Drosophila*.

**Figure 5:**
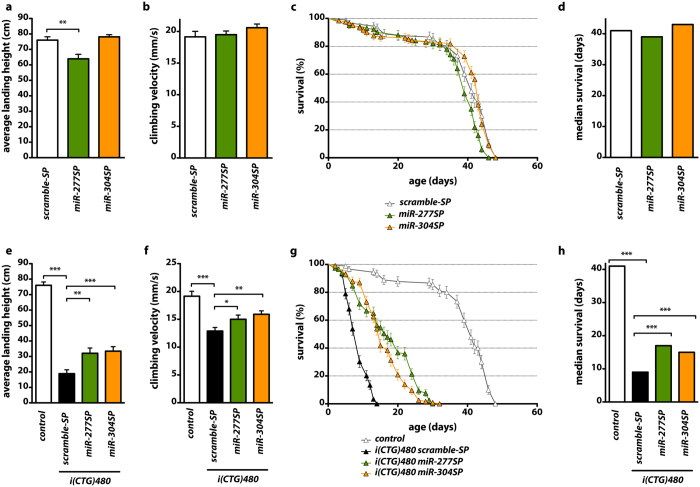
Inhibition of *dme-miR-277* or *dme-miR-304* improved locomotion and survival of DM1 model flies. (**a**,**e**) Average landing height for flies with the indicated relevant genotypes. In control individuals (**a**) *dme-miR-277* silencing decreased landing height while *dme-miR-304* silencing did not affect flight. In DM1 model flies (**e**) expression of *miR-277SP* or *miR-304SP* rescued the reduced flight ability observed. (**b**,**f**) Histograms showing the climbing velocity as mean speed ± SEM in mm/s. In control flies (**b**) silencing of either *dme-miR-277* or *dme-miR-304* had no effect on climbing velocity. However, in DM1 flies (**f**) which have very reduced climbing velocity, expression of *miR-277SP* or *miR-304SP* significantly rescued this phenotype. (**c**,**g**) Survival curves and (**d,h**) median survival, showing that expression of *miR-277SP* or *miR-304SP* had no effect on control but improved survival of DM1 model flies. Between 140 and 160 individuals from each genotype were analyzed. All the indicated transgenes were driven in muscle with *Mhc-Gal4*. *p < 0.05, **p < 0.01, ***p < 0.001 (Student’s t-test).

To assess the correlation between muscle area and locomotor activity we analyzed the flight and climbing ability in flies of different genotypes. Expression of *miR-277SP* in otherwise wild type muscle resulted in a reduction of the average landing height of around 10% in comparison to control flies expressing the *scramble-SP*, which indicates that the reduction of muscle area observed in these flies has a functional correlation (Fig. 5a). However, the muscle atrophy was apparently specific to IFMs since climbing velocity was unchanged in these flies (Fig. 5b). In contrast, silencing of *dme-miR-304* in muscle did not affect locomotor activity of flies (Fig. 5a,b). In DM1 model flies, in comparison to controls not expressing the repeats, concomitant expression of CUG repeats and the *scramble-SP* construct resulted in a drastic reduction of average landing height and climbing velocity (Fig. 5e,f). However, expression of either *miR-277SP* or *miR-304SP* in model flies resulted in a significant partial rescue of both of these parameters to similar levels (Fig. 5e,f). Thus, these results demonstrate that specific silencing of miRNAs regulating *muscleblind* can rescue the muscle atrophy and functional phenotypes characteristic of DM1.

### Functional depletion of *dme-miR-277* or *dme-miR-*304 extends lifespan of DM1 flies

Muscle wasting, particularly in the respiratory system, is the leading cause of death in DM1. We have previously reported that flies expressing *i(CTG)480* in the musculature had a reduced lifespan and median survival compared with control flies^44^. To study whether *dme-miR-277* or *dme-miR-304* silencing rescues lifespan of DM1 flies, we performed survival curves analyses in flies of different genotypes. Importantly, survival curves for flies expressing *miR-277SP* or *miR-304SP* in otherwise wild type muscle were not different to *scramble-SP* control indicating that *dme-miR-277* or *dme-miR-304* silencing did not alter lifespan (Fig. 5c,d). Lifespan of DM1 model flies expressing the *scramble-SP* was significantly reduced compared with control flies that did not express the CTG repeats (Fig. 5g,h). Expression of either *miR-277SP* or *miR-304SP* in model flies increased the median survival. *dme-miR-277* silencing increased median survival by eight days while an increase of six days was detected for DM1 flies expressing *miR-304SP.* Thus, *muscleblind* upregulation triggered by *dme-miR-277* or *dme-miR-304* silencing, improved survival of DM1 model flies. Taken together, our results demonstrate that silencing of specific miRNAs in *Drosophila* triggers an increase of *muscleblind* levels that is sufficient to rescue several molecular and physiological DM1-like features thus supporting miRNA-based derepression of Muscleblind as a potential strategy to treat human DM1.

## Discussion

DM1 presents a considerable disease burden as it is the most common adult-onset muscle dystrophy, and includes cognitive dysfunction, malignant heart arrhythmia, and respiratory failure, ultimately leading to shortened life expectancy. Inhibition of MBNL activity due to sequestration by microsatellite expansion RNAs is a major pathogenic event in DM1. Using a *Drosophila* model of this disease, we confirmed that upregulation of endogenous *muscleblind* by specific microRNA silencing, can rescue DM1-like phenotypes. Endogenous gene modulation to alleviate pathology has been successful in breast cancer where estrogen receptor antagonists are regularly used in clinical practice^46^. Furthermore, pharmacological enhancement of *utrophin* expression, a gene exclusively expressed in fetus but that can compensate Dystrophin loss of function in Duchenne Muscular Dystrophy is currently under investigation^47^. One of the most promising therapeutic strategies for endogenous gene regulation is based on miRNA derepression. These strategies have proven to be beneficial in animal models, to be highly efficient in specific target silencing, and to have appropriate pharmacokinetic parameters to be developed as drugs. In this study we aimed to upregulate endogenous *muscleblind* expression by silencing defined miRNAs, which regulate it in muscle. miRNAs have been extensively associated with several neuromuscular disorders in valuable *in-vivo* systems, which highlights the importance of studying miRNA-based regulation of dystrophy-associated genes as potential therapeutic strategy^48^,^49^,^50^,^51^,^52^. Specifically, we used miRNA sponge constructs, which are transgenes, containing multiple, tandem binding sites of a microRNA of interest, expressed from strong promoters. From our previous studies and TargetScan predictions, we selected a group of microRNAs as potential regulators of *Drosophila muscleblind* and confirmed that sponge constructs for *dme-miR-277* and *dme-miR-304*, which had the highest expression among the sponge constructs tested, reduced the abundance of their respective target miRNAs and achieved upregulation of *muscleblind* at the RNA and protein levels. Isoform-specific quantitative analysis confirmed that each of these sponge construct upregulated different *muscleblind* isoforms and luciferase reporter assays revealed that this regulation was mediated by direct binding of the miRNAs to the 3′ UTR of the different *muscleblind* isoforms. We confirmed direct binding of *dme-miR-277* to the 3′ UTR of the *mbl B* and *D* isoforms and direct binding of *dme-miR-304* to *mbl* isoforms *C* and *D*. Accordingly, the *mblB* and *mblC* isoforms were increased by *miR-277SP* and *miR-304SP*, which reduced the levels of these microRNAs. In the case of *mblD*, we have confirmed direct binding of both microRNAs to its 3′ UTR. Consistently, *miR-304SP* produced an increase of the transcript. However, we have observed a slight but significant decrease of *mblD* transcript as a result of *miR-277SP*, which might be explained by an inter-isoform regulation as *dme-miR-277* also produces a strong upregulation of the *mblB* isoform, which might have a negative effect on *mblD*. Of note, binding sites of *dme-miR-277* and *dme-miR-304* overlap in the *mblD* 3′ UTR, which could explain the difference observed between *in vivo* and luciferase assays for *mblD* regulation by *dme-miR-277*. Interestingly, *miR-304SP* and *miR-277SP* were able to downregulate the expression of *mblB* and *mblC*, respectively, instead of increasing expression, which could also be a consequence of inter-isoform regulation as it has been previously shown for MBNL proteins^53^,^54^.

Since different subcellular localizations, indicative of specific functions, have been reported for different Muscleblind isoforms^38^,^55^, we immunodetected the protein in fly muscle tissue expressing either *miR-277SP* or *miR-304SP*. In both cases we observed Muscleblind overexpression but in different subcellular locations. Whereas *miR-277SP* preferentially increased Muscleblind in sarcomeric bands, *miR-304SP* enhanced Muscleblind expression in nuclei. Consistently, when we analyzed the effects of the sponge constructs on DM1 model flies expressing pathogenic expansions, *miR-277SP* rescued Muscleblind localization in sarcomeric bands and *miR-304SP* increased Muscleblind in nuclei and cytoplasm. Of note, in both cases Muscleblind retention in ribonuclear foci was no longer detectable and protein levels in DM1 flies seemed higher than in normal individuals, suggesting that, in addition to an upregulation of *muscleblind* expression, there might be a release of the protein from foci. Finally, we have shown that MblC localizes to nuclei^55^ and, as we show in this study, is preferentially upregulated by *miR-304SP* expression. Supporting a role for MblC in nuclei, we also confirmed that *miR-304SP* expression rescued a number of Muscleblind-dependent molecular events. Taken together, these data confirm that the upregulation of *muscleblind* achieved by silencing specific regulatory miRNAs is sufficient to rescue critical molecular features that are altered in DM1 model flies.

We also checked the effect of *miR-277SP* and *miR-304SP* expression on muscle atrophy, which is a characteristic DM1 phenotype. We have previously reported that muscle atrophy stems, at least in part, from hyperactivation of the autophagy process and that this had a Muscleblind component. Specifically, we showed that MblC overexpression partially rescued muscle atrophy in the DM1 model flies^27^ and, consistent with these previous observations, we confirmed that *miR-304SP* expression in model flies also rescued muscle atrophy. However, processes other than splicing must be involved in triggering this phenotype as *miR-277SP* expression, which preferentially upregulates Muscleblind in sarcomeric bands, was also able to rescue muscle area in the DM1 model flies. Importantly, the increase in IFM muscle area achieved by expression of sponge constructs had a functional correlation, as survival, climbing and flight abilities also improved.

By expressing the sponge constructs with the *Mhc-Gal4* driver we also tested the effects of long-term Muscleblind overexpression. In control flies, we observed that *miR-304SP* expression, caused a 6-fold increase in *muscleblind* relative expression and had no effect on muscle area, survival or locomotor function. However, *miR-277SP* expression, which produced a 15-fold upregulation of *muscleblind* caused a significant reduction in muscle area, which correlated with decreased landing height. In a CTG expressing background, however, expression of either of the sponge constructs brought about beneficial effects suggesting that limited overexpression of additional natural miRNA target transcripts are negligible compared to the positive effects of boosting *muscleblind*. Previous studies have confirmed that long-term overexpression of *MBNL1* in mouse models is well tolerated when limited to skeletal muscle. *MBNL1* overexpression, in a range of 10 to 17 fold, caused no detectable histopathology or functional abnormalities^28^. Deletereous effects of *miR-277SP* could originate from overexpression of several targets in addition to *musclebind*, as *dme-miR-277* is one of the miRNAs with highest expression in muscle^35^. Importantly, we show for the first time functional locomotor defects in DM1 model flies expressing CUG repeats in skeletal muscle and, according to our data, flight assays seem more sensitive than climbing assays as small differences in muscle area translate into detectable differences in flight ability.

Conceptually similar to sponge constructs, several drugs targeting specific miRNAs are currently being developed for the treatment of human diseases. Miravirsen, a drug targeting *hsa-miR-122*, the hepatocyte-specific microRNA that Hepatitis C virus hijacks and uses to self-replicate^56^, is one the most advanced. Our study with the *Drosophila* model of DM1 sets the stage for the evaluation of miRNA blockers to de-repress *muscleblind* expression as a valid and powerful therapeutic target for treatment of DM1.

## Materials and Methods

### *Drosophila* stocks

*Mhc-Gal4* flies were described in ref. 57. miRNA sponge lines (*UAS-miR-SP*) for *dme-miR-92a*, *dme-miR-100*, *dme-miR-124*, *dme-miR-277, dme-miR-304* and *scramble-SP* (control) were obtained from Dr. T. Fulga^35^. Briefly, *miR-SP* constructs were designed with a silencing cassette of 20 repetitive miRNA complementary sequences separated by variable four-nucleotide linker sequences. The recombinant line *Mhc-Gal4 UAS-i(CTG)480* was generated in ref. 42. *UAS-mblC* flies^58^ and *UAS-IR-mbl* flies^42^ were previously reported. All crosses were carried out at 25 °C with standard fly food. Transgene doses were the same for control and experimental conditions throughout this work.

### RNA extraction, RT-PCR and qRT-PCR

For each biological replicate, total RNA from 10 adult males was extracted using Trizol (Sigma). One microgram of RNA was digested with DNaseI (Invitrogen) and reverse-transcribed with SuperScript II (Invitrogen) using random hexanucleotides. 20 ng of cDNA were used in a standard PCR reaction with GoTaq polymerase (Promega) and specific primers were used to analyze *Fhos* exon 16′ and *Tnt* exon 3–5 splicing (Table S2). *Rp49* was used as endogenous control using 0.2 ng of cDNA. qRT-PCR was carried out on 2 ng of cDNA template with SYBR Green PCR Master Mix (Applied Biosystems) and specific primers (Table S1). For reference gene, *Rp49*, qRT-PCR was carried out on 0.2 ng of cDNA. Thermal cycling was performed with Step One Plus Real Time PCR System (Applied Biosystems). Three biological replicates and three technical replicates per biological sample were carried out. Relative expression to endogenous gene and the control group was obtained by the 2^−∆∆Ct^ method. Pairs of samples were compared using two-tailed t-test (α = 0.05), applying Welch’s correction when necessary.

### Luciferase reporter assay

A luciferase assay using the pEZX-MT05 vector was performed to validate the binding of *dme-miR-277* and *dme-miR-304* to *mblA, mblB, mblC,* and/or *mblD* 3′ UTR regions. pEZX-MT05 vector contains a secreted Gaussia luciferase (GLuc) ORF driven by SV40 promoter as a reporter of the 3′ UTR expression and a secreted Alkaline Phosphatase (SEAP) reporter driven by a CMV promoter as an internal control. Firstly, the 3′ UTR *mbl* isoforms were generated by amplifying genomic *Drosophila* DNA with specific primers using Pfu DNA polymerase (Biotools) (Table S1). The PCR products were cloned into the *Eco*RV site of pBluescript II KS vector. The plasmids containing the 3′ UTR *mbl* isoforms were cut out and subcloned into the pEZX-MT05 vector at the *Xho*I and *Sfa*AI sites. The constructs were verified by sequencing the plasmids from both ends. Secondly HeLa cells were used for the 3′ UTR luciferase assay. Cells were maintained in DMEM supplemented with 10% FBS and 1% penicillin-streptomycin at 37 °C and 5% CO_2_. HeLa cells were seeded (5 × 10^4^/well) in 24-well plates. A total of 500 ng/well of pEZX-MT05 vector containing *mblA, mblB, mblC* or *mblD* were cotrasfected with 500 ng/well of pCMVMIR vector (Blue Heron) containing *dme-miR-277* or *dme-miR-304*, using X-tremeGENE TM HP DNA Transfection Reagent (Sigma-Aldrich). 48 h and 72 h after transfection, Gaussia luciferase (GLuc) and alkaline phosphatase (SEAP) activities were measured by luminescence in conditioned medium using the secreted-pair dual luminescence kit (GeneCopoeia). Gaussia luciferase activity was normalized to alkaline phosphatase activity (GLuc/SEAP). The statistical differences were analyzed using the Student’s t test (p < 0.05) on normalized data.

### MicroRNA quantification

*UAS-miR-277SP, UAS-miR-304SP and UAS-Scramble-SP* were expressed in flies under the control of a muscle specific *Mhc-Gal4* driver. Total RNA from thoraces was isolated according to the miRNeasy miRNA kit protocol without enrichment for miRNAs (Qiagen). The expression analysis of *dme-miR-277* and *dme-miR-304* was performed by real-time PCRs with specific miRCURY LNA microRNA PCR primers (Exiqon) according to the manufacturer’s instructions. As reference genes we used *dme-miR-7* and *dme-miR-8*. Expression level determinations were performed using an Applied Biosystems Step One Plus Real Time PCR System and the values were calculated using the 2^−∆∆Ct^.

### Western blotting

For total protein extraction 20 female thoraces were homogenized in RIPA buffer (150 mM NaCl, 1.0% IGEPAL, 0.5% sodium deoxycholate, 0.1% SDS, 50 mM Tris-HCl pH 8.0) plus protease and phosphatase inhibitor cocktails (Roche Applied Science). Total proteins were quantified with BCA protein assay kit (Pierce) using bovine serum albumin as standard. 20 μg of samples were denatured for 5 min at 100 °C, resolved in 12% SDS-PAGE gels and transferred onto polyvinylidene difluoride (PVDF) membranes. The membranes were blocked with 5% nonfat dried milk in PBS-T (8 mM Na_2_HPO4, 150 mM NaCl, 2 mM KH_2_PO4, 3 mM KCl, 0.05% Tween 20, pH 7.4) and immunodetected following standard procedures. For Mbl protein detection anti-Mbl antibody^40^ was pre-absorbed against early stage wild type embryos (0–6 h after egg laying) to eliminate non-specific binding of antibody. Membranes were incubated with pre-absorbed primary (overnight, 1:1000) followed by horseradish peroxidase (HRP)-conjugated anti-sheep-IgG secondary antibody (1 h, 1:5000, Sigma-Aldrich). Loading control was anti-Tubulin (overnight, 1:5000, Sigma-Aldrich) followed by incubation with HRP-conjugated anti-mouse-IgG secondary antibody (1 h, 1:3000, Sigma-Aldrich). Bands were detected using ECL Western Blotting Substrate (Pierce). Images were acquired with an ImageQuant LAS 4000 (GE Healthcare).

### Histological analysis

Inmunofluorescence detection of Muscleblind in fly muscle and analysis of the IFM area in *Drosophila* thoraces were performed as previously described^42^. Briefly, six adult female thoraces were embedded in Epon following standard procedures. After drying the resin, semi-thin sections of 1.5 μm were obtained using an ultramicrotome (Ultracut E, Reichert-Jung and Leica). Images were taken at 100× magnification with a Leica DM2500 microscope. To quantify muscle area, sections were counterstained with toluidine blue and five images containing IFMs per fly were converted into binary images. Considering the complete image as 100% of the area, we used NIH ImageJ software to calculate the percentage occupied by pixels corresponding to IFMs. P-values were obtained using a two-tailed, non-paired t-test (α = 0.05), applying Welch’s correction when necessary.

### *Drosophila* lifespan analyses

A total of 120 newly hatched flies with the appropriate genotypes were collected and kept at 29 °C. Flies were transferred to new fresh nutritive media every second day and the decline in number was scored on a daily basis. Survival curves were obtained using the Kaplan-Meier method and statistical analysis was performed with a log-rank (Mantel-Cox) test (α = 0.05) using the GraphPad Prism5 software.

### Functional assays

Flight assays were performed at day 5 according to ref. 59 using 100 male flies per group. Landing distance was compared between groups using two-tailed t-test (α = 0.05). To assess climbing velocity groups of ten 5-day-old males were transferred into disposable pipettes (1.5 cm in diameter and 25 cm height) after a period of 24 h without anesthesia. The height reached from the bottom of the vial by each fly in a period of 10 s was recorded with a camera. For each genotype, two groups of 30 flies were tested. Two-tailed t-test (α = 0.05) was used for comparisons of pairs of samples applying Welch’s correction whenever necessary.

## Additional Information

**How to cite this article**: Cerro-Herreros, E. *et al.* Derepressing *muscleblind* expression by miRNA sponges ameliorates myotonic dystrophy-like phenotypes in *Drosophila. Sci. Rep.* **6**, 36230; doi: 10.1038/srep36230 (2016).

**Publisher’s note:** Springer Nature remains neutral with regard to jurisdictional claims in published maps and institutional affiliations.

## Supplementary Material

Supplementary Information

## Figures and Tables

**Table 1:** Number of miRNA recognition sites predicted in *muscleblind* 3′ UTR according to different algorithms.

miRNA	Miranda	TargetScan	*mbl* isoform
*miR-92a*	—	—	*mblA*
	—	—	*mblB*
	—	—	*mblC*
	—	—	*mblD*
*miR-100*	—	—	*mblA*
	—	—	*mblB*
	—	—	*mblC*
	—	—	*mblD*
*miR-124*	1 site	—	*mblA*
	—	—	*mblB*
	—	—	*mblC*
	1 site	—	*mblD*
*miR-277*	1 site	—	*mblA*
	2 sites	—	*mblB*
	—	—	*mblC*
	2 sites	1 site	*mblD*
*miR-304*	—	—	*mblA*
	—	—	*mblB*
	1 site	—	*mblC*
	—	1 site	*mblD*

-: no predictions.
