# Supplementary material for: Derepressing muscleblind expression by miRNA sponges ameliorates myotonic dystrophy-like phenotypes in Drosophila
Source: Sci Rep. 2016 Nov 2;6:36230. doi: 10.1038/srep36230 (PMC5090246; doi:10.1038/srep36230)
Supplement: Supplementary Information [file srep36230-s1.pdf]

Derepressing *muscleblind* expression by miRNA sponges ameliorates myotonic dystrophy-like phenotypes in *Drosophila*.

Estefania Cerro-Herreros[1,2], Juan M. Fernandez-Costa[1,2], María Sabater-Arcis [1,2], Beatriz Llamusi[1,2] and Ruben Artero[1,2]*

Supplementary Information

Fig.S1. Quantification of the expression of the mCherry reporter contained in the SP constructs. The graph represents the fluorescence intensity of mCherry protein (RFU) normalized to total protein. *miR-277SP*, *miR-304SP* and scramble-SP were the three SPs with the highest expression levels in flies, while *miR-92aSP*, *miR-100SP* and *miR-124SP* showed lower expression of the reporter. Dotted line indicates background levels (control flies expressing no reporter).

Fig. S2. MblC regulates alternative splicing of *Fhos* e16 and *Serca* e13, and *CyP6W1* expression. (**a** and **d**) Semiquantitative RT-PCR showing splicing of *Fhos* exon 16 (**a**) and Tnt exon 3-5 splicing (**d**) in flies with the indicated relevant genotypes. *Rp49* transcripts were detected as endogenous control. (**b**) Quantification of percentage of inclusion of Fhos exon 16. Aberrant e16 inclusion in DM1 flies (*i(CTG)480*), was rescued by MblC expression (*i(CTG)480, MblC*). Moreover, silencing of Mbl in skeletal muscle (*IR-mbl*) caused a significant exon 16 inclusion while MblC overexpression in a wild type background (*MblC*) promotes the exclusion of *Fhos* exon 16. In contrast, neither CTG repeats nor MblC had any effect on exon 3-5 *Tnt* splicing (**d** and **e**). (**c** and **f**) Quantification of *Serca* exon 13 and *Cyp6W1* expression levels by qRT-PCR. CTG expansions (*i(CTG)480*) triggered the exclusion of *Serca* exon 13 whilst MblC promoted exon inclusion (**c**). Furthermore, *Cyp6W1* expression level was increased in DM1 flies (*i(CTG)480*) while MblC overexpression promoted the reduction of this expression (**f**). (**g**) Outline of the intron/exon structure of relevant splicing events for *Fhos, Tnt and Serca* genes. Arrows denote primers used for semi and quantitative RT-PCR analyses. Red and green lines

indicate alternative splicing events**.** Expression of all indicated transgenes was targeted to muscle with *Mhc-Gal4*. *p<0.05, **p<0.01, ***p<0.001 (Student's t-test).

mCherry quantification

UAS-miR-92aSP, UAS-miR-100SP, *UAS-miR-124SP, UAS-miR-277SP,* and *UAS-mir-304SP* were expressed in flies under the control of muscle specific *Mhc-Gal4* driver. These flies contain SP elements, which comprise 20 miRNA binding sites in the 3′-untranslated region of mCherry under the control of 10 tunable Gal4 UAS binding sites. 10 female flies were collected and homogenized in 50 mM Tris-HCl pH 8.0 buffer. 100 µL of homogenate were added to a black 96-well plate and fluorescence was measured. mCherry was recorded using an excitation wavelength/bandwidth of 530/20 nm and emission wavelength/bandwidth of 590/40 nm with a Fluoroskam Ascent FL microplate reader (Thermo Scientific). Protein levels in fly lysates were determined with BCA assay (Pierce), using Tecan Infinite 200 PRO (Life Sciences), and were used to normalize red fluorescence levels. Three independent measurements were taken for each genotype.

Fig. S1

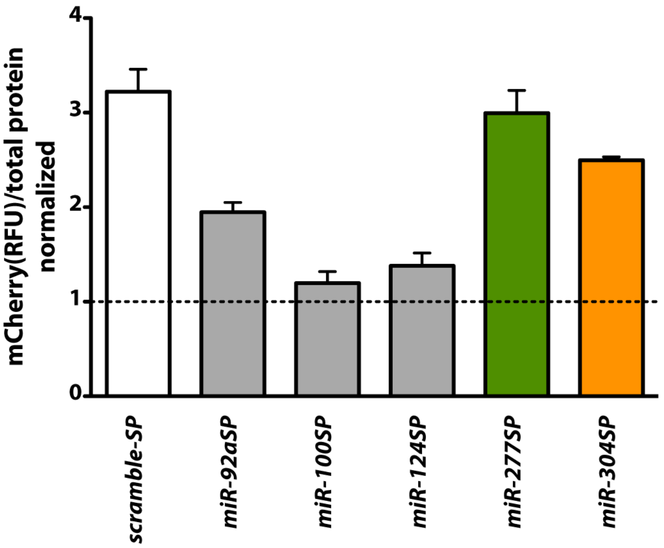

mCherry(RFU)/total protein
normalized
4
3
2
1
0
scramble-SP
miR-92aSP
miR-100SP
miR-124SP
miR-277SP
miR-304SP

Fig. S2

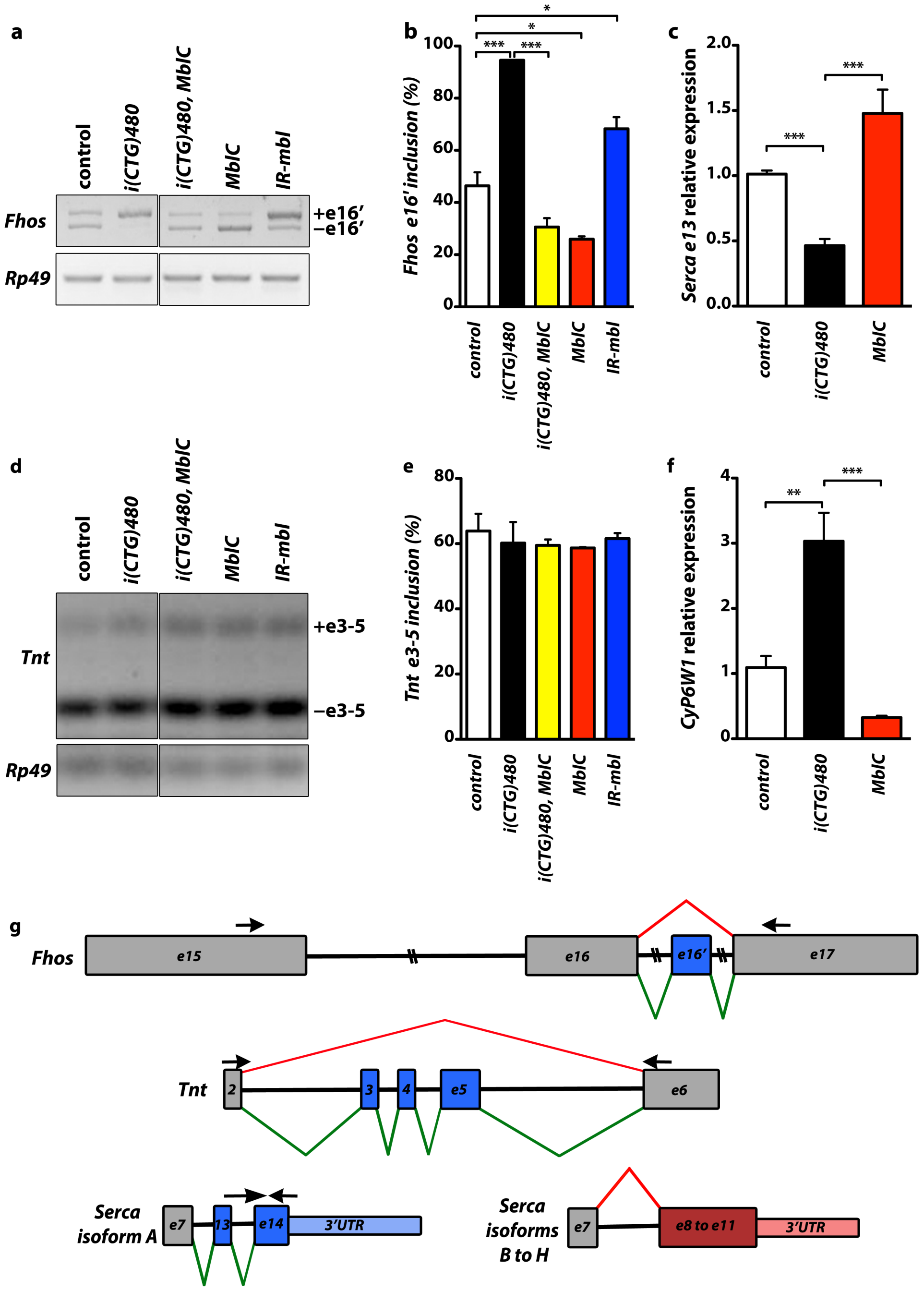

a
control
i(CTG)480
i(CTG)480, MblC
MblC
IR-mbl
Fhos
+e16′
−e16′
Rp49
b
Fhos e16′ inclusion (%)
100
80
60
40
20
0
***
***
*
*
control
i(CTG)480
i(CTG)480, MblC
MblC
IR-mbl
c
Serca e13 relative expression
2.0
1.5
1.0
0.5
0.0
***
***
control
i(CTG)480
MblC
d
control
i(CTG)480
i(CTG)480, MblC
MblC
IR-mbl
Tnt
+e3-5
−e3-5
Rp49
e
Tnt e3-5 inclusion (%)
80
60
40
20
0
control
i(CTG)480
i(CTG)480, MblC
MblC
IR-mbl
f
CyP6W1 relative expression
4
3
2
1
0
**
***
control
i(CTG)480
MblC
g
Fhos
e15
e16
e16′
e17
Tnt
2
3
4
e5
e6
Serca isoform A
e7
13
e14
3′UTR
Serca isoforms B to H
e7
e8 to e11
3′UTR

Table S1. qPCR raw data

	**Sample**	**Target**	**C_T Mean**	**Target**	**C_T Mean**
Mhc-Gal4 x UAS-SP	Scramble-SP	*mbl*	28.63888741	*rp49*	28.78798612
	miR-92SP		25.82085864		26.70564842
	miR-100SP		26.09071255		26.05283455
	miR-124SP		26.85871792		27.18436635
	miR-277SP		24.54497973		28.51061058
	miR-304SP		24.04358292		26.84158007
	Sample	Target	C_T Mean	Target	C_T Mean
Mhc-Gal4 UAS-i(CTG)480 x UAS-SP	Scramble-SP	*mbl*	23.29541016	*rp49*	24.09894867
	miR-277SP		22.7860775		27.19969304
	miR-304SP		23.39365069		26.32800992

	**Sample**	**Target**	**C_T Mean**	**Target**	**C_T Mean**
Mhc-Gal4 x UAS-SP	Scramble-SP	*mbl A*	25.47866313	*rp49*	21.98299281
	miR-277SP		26.24561691		21.87879372
	miR-304SP		24.40922228		21.60143153
	Scramble-SP	*mbl B*	26.17312686		21.98299281
	miR-277SP		24.52533404		21.87879372
	miR-304SP		26.66050148		21.60143153
	Scramble-SP	*mbl C*	22.54533323		21.98299281
	miR-277SP		23.40289688		21.87879372
	miR-304SP		21.3717734		21.60143153
	Scramble-SP	*mbl D*	26.88251686		21.98299281
	miR-277SP		27.72441228		21.87879372
	miR-304SP		25.43103663		21.60143153

Table S2. Oligonucleotide sequences

Primer	Sequence (5’ → 3’)	qRT-PCR/RT-PCR
mbl fwd	TTGAATCAAAATTATAGCCCAAGCT	qRT-PCR
mbl rev	CGATTTTGCTCGTTAGCGTTT	qRT-PCR
mblA fwd	CAGACACCGAAATACTCTCTACAAACA	qRT-PCR
mblA rev	AAAATCAGGAGTAAACAAATACACGTAGAC	qRT-PCR
mblB fwd	CACACATCCAGATATGCTACTTACCA	qRT-PCR
mblB rev	TGAGCGATTTCGATTGATTTTG	qRT-PCR
mblC fwd	CAGCAAACACACATCACCTACCA	qRT-PCR
mblC rev	CTATCGAGCAGGAGGATGAAGAG	qRT-PCR
mblD fwd	GCCTCTGGAAAATGCTGCAA	qRT-PCR
mblD rev	CAGCAACCGCAAAAGAGCTT	qRT-PCR
Serca fwd	GCAGATGTTCCTGATGTCG	qRT-PCR
Serca rev	CGTCCTCCTTCACATTCAC	qRT-PCR
Cyp6w1 fwd	TTGCGCACAAAAATCTCTCC	qRT-PCR
Cyp6w1 rev	GTCCTGCAAGTTCTTTCCAA	qRT-PCR
Rp49 fwd	GGATCGATATGCTAAGCTGTCGCACA	qRT-PCR/ RT-PCR
Rp49 rev	GGTGCGCTTGTTCGATCCGTAACC	qRT-PCR/ RT-PCR
Fhos fwd	GTCATGGAGTCGAGCAGTGA	RT-PCR
Fhos rev	TGTGATGCGGGTATCTACGA	RT-PCR
Tnt fwd	CGACGATGAAGAGTACAC	RT-PCR
Tnt rev	ACTCGGTGATGTATTCTTTCAG	RT-PCR
mblA 3'UTR fwd	CAGCGATCGCCGCGATTTTATTTACGCTTAC	PCR
mblA 3'UTR rev	CACTCGAGGTTTAGTGGTTAGAGCCGA	PCR

mblB 3'UTR fwd	CCGCGATCGCTTTGTTATCCTCATTCCTTTG	PCR
mblB 3'UTR rev	CGCGCTCGAGGATCGGTTTTATAATTTTTG	PCR
mblC 3'UTR fwd	GGCTCGAGACCAGAGACGTATATTA	PCR
mblC 3'UTR rev	AGCGATCGCTAAGTTGTTATTAATAC	PCR
mblD 3'UTR fwd	GCGATCGCCTAATTAATTATGCAGTA	PCR
mblD 3'UTR rev	CTCGAGTCGACTTCAATTTCGAAT	PCR
